# Protection by Face Masks against Influenza A(H1N1)pdm09 Virus on Trans-Pacific Passenger Aircraft, 2009

**DOI:** 10.3201/eid1909.121765

**Published:** 2013-09

**Authors:** Lijie Zhang, Zhibin Peng, Jianming Ou, Guang Zeng, Robert E. Fontaine, Mingbin Liu, Fuqiang Cui, Rongtao Hong, Hang Zhou, Yang Huai, Shuk-Kwan Chuang, Yiu-Hong Leung, Yunxia Feng, Yuan Luo, Tao Shen, Bao-Ping Zhu, Marc-Alain Widdowson, Hongjie Yu

**Affiliations:** Chinese Center for Disease Control and Prevention, Beijing, China (L. Zhang, Z. Peng, G. Zeng, M. Liu, F. Cui, H. Zhou, Y. Feng, Y. Luo, T. Shen, B.-P. Zhu, H. Yu);; Fujian Center for Disease Control and Prevention, Fuzhou, China (J. Ou, R. Hong); Nanchang Center for Disease Control and Prevention, Nanchang, China (M. Liu);; Centers for Disease Control and Prevention, Atlanta, Georgia, USA (R. E. Fontaine, M.-A. Widdowson);; China–US Collaborative Program on Emerging and Re-emerging Infectious Diseases, Beijing (Y. Huai);; Hong Kong Department of Health, Hong Kong, China (S.-K. Chuang, Y.-H. Leung)

**Keywords:** influenza, influenza A virus, influenza A(H1N1)pdm09 virus, viruses, aircraft, trans-pacific passenger aircraft, disease outbreak, face masks, China, Hong Kong, United States, outbreak, air travel, travel

## Abstract

In response to several influenza A(H1N1)pdm09 infections that developed in passengers after they traveled on the same 2 flights from New York, New York, USA, to Hong Kong, China, to Fuzhou, China, we assessed transmission of influenza A(H1N1)pdm09 virus on these flights. We defined a case of infection as onset of fever and respiratory symptoms and detection of virus by PCR in a passenger or crew member of either flight. Illness developed only in passengers who traveled on the New York to Hong Kong flight. We compared exposures of 9 case-passengers with those of 32 asymptomatic control-passengers. None of the 9 case-passengers, compared with 47% (15/32) of control-passengers, wore a face mask for the entire flight (odds ratio 0, 95% CI 0–0.71). The source case-passenger was not identified. Wearing a face mask was a protective factor against influenza infection. We recommend a more comprehensive intervention study to accurately estimate this effect.

After influenza A(H1N1)pdm09 virus was identified in April 2009 (*1*), it spread rapidly, largely through air travel by infected passengers (*2*). On May 2, 2009, China implemented intensive screening of arriving air passengers by using thermal cameras to detect fever and a short questionnaire about existing respiratory symptoms and fever; passengers were advised to seek medical consultation if fever or respiratory symptoms developed ≤7 days of arrival (*3**,**4*). Nasopharyngeal swab specimens collected from all arriving febrile passengers were tested for virus at the nearest provincial, city, or county Centers for Disease Control (CDC) laboratory by using real-time reverse transcription PCR (RT-PCR) (*5*). If any of these results were positive results, all passengers on the same flight were quarantined.

On May 11, 2009, this system detected the first confirmed influenza A(H1N1)pdm09 infection in mainland China in a US traveler (*6*). As of May 29, the system detected 21 other imported infections in passengers arriving on international flights. On May 29, the first locally acquired influenza A(H1N1)pdm09 infection was detected.

On May 30, acute onset of fever (38.3°C), cough, sore throat, and headache developed in a 22-year-old man. He sought treatment at a clinic in Fuzhou, China, where medical staff learned that he recently arrived from New York, New York, USA (hereafter referred to as New York) and reported a suspected case of influenza A(H1N1)pdm09 infection to the county CDC. On May 31, duplicate nasopharyngeal swabs specimens from the patient were positive for influenza A(H1N1)pdm09 virus at Fuzhou CDC and Fujian Provincial CDC.

On May 27 at 10:40 am (all times Beijing local time), the patient had departed New York on a flight to Hong Kong, China. After flying for 5 hours and 50 min, the plane made a scheduled stopover in Vancouver, British Columbia, Canada. All passengers remained on board during the stopover, which lasted 1 hour and 15 min (4:30 pm–5:45 pm). Air-handling systems were fully operational. The aircraft left Vancouver and flew for 13 hours and 15 min and arrived in Hong Kong at 7:00 am on May 28. In Hong Kong, 63 passengers transferred to a Hong Kong to Fuzhou flight, which departed Hong Kong at 8:50 am and arrived at in Fuzhou City Airport at 10:30 am (flight time 1 hour and 40 min.).

The aforementioned patient had no fever or respiratory symptoms when screened on arrival in Fuzhou. The Fujian Provincial CDC, concerned that other passengers on the Hong Kong to Fuzhou flight might be infected, traced and quarantined (involuntary social distancing) the arriving passengers and crew members in their own homes, designated hotels, or hospitals. According to Chinese Ministry of Health guidelines (*7*), social contacts of this confirmed case-patient were traced and quarantined. These passengers, crew members, and contacts were monitored for 7 days for fever and respiratory illness; nasopharyngeal swab specimens were obtained from symptomatic persons. This effort identified 7 additional case-passengers on the Hong Kong to Fuzhou flight in whom symptoms developed during May 30–June 1 and had influenza A(H1N1)pdm09 infection confirmed by RT-PCR. All 8 case-passengers had arrived in Hong Kong on the same New York to Hong Kong flight. The China CDC and Fujian Provincial CDC initiated an outbreak investigation to assess possible transmission of influenza A(H1N1)pdm09 virus on those flights and better understand risks for influenza spread in confined settings.

## Methods

### Case Definition

We defined a suspected case of influenza A(H1N1)pdm09 infection as an acute, febrile respiratory illness with onset during May 21–June 4, 2009, among passengers or crew members on the New York to Hong Kong flight on May 27 or the Hong Kong to Fuzhou flight on May 28. A confirmed case was a suspected case with laboratory evidence of influenza A(H1N1)pdm09 infection by PCR testing of respiratory specimens (*5*). We defined influenza-like illness (ILI) as acute onset of fever (≥37.5°C) and cough or sore throat.

### Retrospective Investigation

From the Fuzhou airport quarantine post, we obtained a list of passengers who arrived in Fuzhou on the Hong Kong to Fuzhou flight. All passengers had been quarantined for 7 days at home or in designated hotels or hospitals. Body temperatures were measured daily; if fever (≥37.5°C) or respiratory symptoms developed in passengers, a nasopharyngeal swab specimen was obtained and tested for influenza A(H1N1)pdm09 by using RT-PCR. Health professionals at the Centre for Health Protection, Department of Health, Hong Kong, attempted to contact all passengers on the New York to Hong Kong flight who had disembarked in Hong Kong. These professionals obtained information from these passengers regarding onset of fever and respiratory symptoms, medical care, antiviral drugs, and underlying medical conditions. We were unable to contact passengers who transferred to connecting flights from Hong Kong to other destinations in China or Southeast Asia.

**Figure 1:**
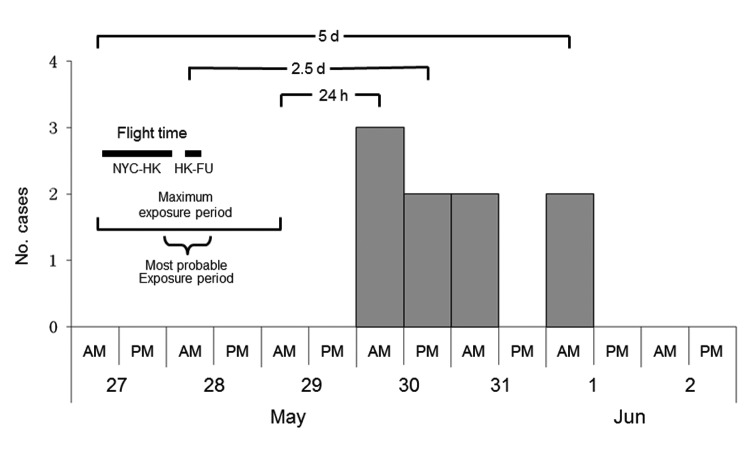
Time of disease onset for persons infected with influenza A(H1N1)pdm09 virus on an international flight from New York, New York (NYC), to Hong Kong (HK) and Fujian Province (FU), China, May 2009. The most probable exposure period was calculated by subtracting median incubation time for influenza A(H1N1)pdm09 (2.5 days) from the time interval containing median onset of a case (pm, May 30). Beginning of the maximum exposure period was calculated by subtracting the maximum incubation period (5 days) from the midpoint of the interval containing onset of the most recent case (am, June 1). End of the maximum exposure period was calculated by subtracting the minimum incubation period (24 h) from the midpoint of the interval containing onset of first case (am, May 30).

To approximate the most probable exposure period for this apparent point-source outbreak (Figure 1), we subtracted the median incubation period for influenza A(H1N1)pdm09 (2.5 days) (*8**–**11*) from the 12-hour interval for onset of the median case (pm, May 30) (*12*). To approximate the beginning of the maximum exposure period, we subtracted the maximum incubation period (5 days) from the midpoint of the interval for onset of the most recent case (am, June 1). Similarly, for the end of the maximum exposure period, we subtracted the minimum incubation period (24 hours) from the midpoint of the interval containing onset of the first case (am, May 30). We compared attack rates by flight and examined aircraft seating charts for spatial distribution of case-passengers and their mutual proximity.

### Case–Control Study

To assess risk factors for transmission of influenza A(H1N1)pdm09 on the New York to Hong Kong flight, we conducted a case–control study. We compared exposure history and other risk factors of 9 confirmed case-passengers with those of 32 control-passengers in the economy-class cabin. We attempted to contact 55 noninfected passengers who disembarked in Fuzhou and 18 noninfected passengers who disembarked in Hong Kong, and we interviewed all persons >5 years of age who agreed to be interviewed. Crew members and business-class passengers were excluded. A total of 32 noninfected passengers provided complete information and served as controls. Of these 32 control-passengers, 28 boarded in New York; 27 disembarked in Fuzhou and 1 disembarked in Hong Kong; and 4 boarded in Vancouver and disembarked in Hong Kong.

We conducted face-to-face interviews with case- and control-passengers bound for Fuzhou at hospitals or hotel rooms where they were quarantined. For passengers quarantined at home or who disembarked in Hong Kong (including 1 case-passenger in Hong Kong), interviews were conducted by telephone. Using a standard questionnaire, we interviewed case- and control-passengers on factors potentially affecting the likelihood of influenza A(H1N1)pdm09 virus infection during the 7 days before and during the flight. These factors included contact with ILI patients ≤1 week before the flight, moving around the airplane during the flight, lavatory use, handwashing, face mask use (wearing a face mask, for how long, and when they wore it and did not wear it), and talking with other passengers.

### Laboratory Testing

Respiratory specimens (nasal, throat, and nasopharyngeal swab specimens and nasopharyngeal aspirates) were collected from suspected case-passengers and persons being quarantined in whom fever or respiratory symptoms developed. We detected influenza A(H1N1)pdm09 virus nucleic acid by using RT-PCR and standard PCR with virus-specific primers according to standard protocols (*5**,**13**,**14*) at biosafety level 2 laboratories at the Fuzhou CDC, the Fujian CDC, and the Public Health Laboratory Centre at the Hong Kong Department of Health.

### Statistical Analysis

We used Fisher exact test to compare frequencies between case and control groups and StatXact 8 (*15*) to calculate exact odds ratios (ORs), 95% CIs, and p values. All statistical tests were 2-sided and had a power of α = 0.05.

## Results

### Outbreak Description

Of 144 persons (136 passengers and 8 crew) on the Hong Kong to Fuzhou flight, follow-up and quarantine measures were completed for 140; 8 (5.7%) had confirmed influenza A(H1N1)pdm09 infections; all 8 had ILI. Four additional febrile passengers did not have respiratory symptoms and were negative for influenza A(H1N1)pdm09 virus. In addition, 3 (7.5%) of 40 social contacts of case-passengers had ILI; 2 had confirmed influenza A(H1N1)pdm09 infections. All 8 confirmed case-passengers with influenza A(H1N1)pdm09 infections were among 63 passengers who had transferred from the New York to Hong Kong flight (attack rate 13%), compared with none among 73 other passengers who boarded in Hong Kong (attack rate 0) (p<0.01, by Fisher exact test) or among the 8 crew members. The investigation focused on the New York to Hong Kong flight. All 9 (8 in Fuzhou and 1 in Hong Kong) case-passengers had departed on the New York to Hong Kong flight at 10:40 am on May 27 (total flight time 20 hours and 20 min).

A total of 260 passengers and 14 crew members were on the New York to Hong Kong flight. After arrival in Hong Kong, 63 passengers transferred to the Hong Kong to Fuzhou flight, 91 passengers disembarked at Hong Kong, and 106 passengers transferred to flights bound for other cities in China or Southeast Asia. The Centre for Health Protection at the Hong Kong Department of Health traced 19 of 91 passengers who disembarked in Hong Kong. One (5.3%) had ILI and a confirmed influenza A(H1N1)pdm09 infection. The attack rate for the 63 Fuzhou passengers and 19 Hong Kong passengers who could be evaluated was 11% (9/82). Among 72 passengers who disembarked in Hong Kong but could not be contacted, some probably continued traveling in China by bus, ferry, train, and car. We were unable to trace the 106 passengers who disembarked in Hong Kong and flew to other destinations.

**Figure 2:**
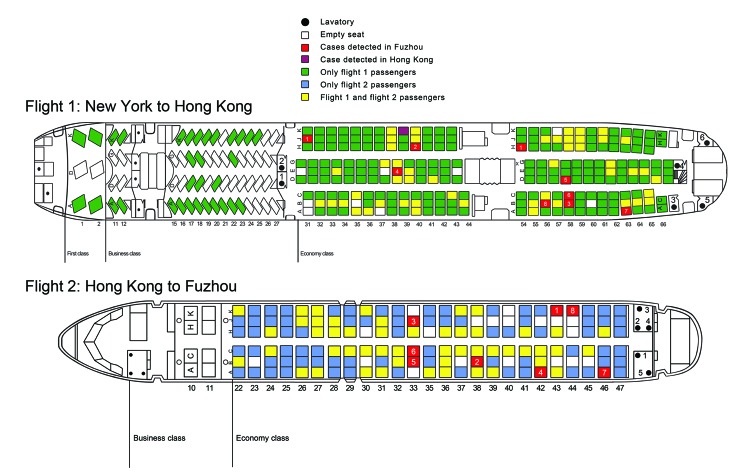
Schematic diagrams of the plane for the flight from New York, New York, to Hong Kong, China (Flight 1), and the plane for the flight from Hong Kong to Fuzhou, China, (Flight 2), May 2009. Case-passenger 1 on the flight from New York to Hong Kong changed his seat in Vancouver, British Columbia, Canada.

All 9 infected passengers had mild, self-limiting ILI characterized by fever (100%) and cough (78%) or sore throat (44%). Onset of fever or respiratory illness occurred during May 30–June 1 (3 days) (median onset time during the second 12 hours of May 30), suggesting a point source (Figure 1). Using the 2.5-day median incubation periods for influenza A(H1N1)pdm09, the most probable exposure period was from midnight to noon on May 28, which coincides with the final 6 hours of the New York to Hong Kong flight, waiting in the Hong Kong airport, and during the Hong Kong to Fuzhou flight. The maximum estimated exposure period for this point-source outbreak was from 12 hours before departure from New York to 12 hours after arrival in Fuzhou. Case-passengers sat throughout economy-class cabins on the New York to Hong Kong flight (Figure 2). Age range of the 9 infected passengers (5 male passengers) was 6–46 years (median 20 years).

All 144 passengers and crew members on the Hong Kong to Fuzhou flight and the 91 passengers and crew members on the New York to Hong Kong flight were screened for fever and respiratory symptoms at arrival at Fuzhou Airport. The other 106 passengers who flew to other cities in China or Southeast Asia were not screened in Hong Kong. One passenger on arrival in Fuzhou had fever (37.5°C) and a stuffy nose, but duplicate nasopharyngeal specimens were negative for influenza A(H1N1)pdm09 virus, and repeat temperature checks showed no fever (37.2°C). Three days later, ILI abruptly developed in this passenger (temperature 38.5°C), and infection with influenza A(H1N1)pdm09 virus was confirmed. This passenger and 2 contacts in New York (father and a co-worker) had nasal congestion without fever or ILI since May 16. No other case-passenger recalled recent respiratory illness before or during the flights or contact with any person with respiratory illness during the week before departure or with another passenger who had respiratory illness during either flight or after arriving in Fuzhou. During the 5 days before onset, 1 person had taken another flight and 1 had visited a tourism site (Chinatown) in New York.

### Case–Control Investigation

Children were underrepresented in the control group, but age and sex of these children did not differ (Table). From New York to Vancouver, 11% (1/9) case-passengers wore a face mask compared with 57% (16/28) of control-passengers (OR 0.094, 95% CI 0.002–0.91). From Vancouver to Hong Kong, no case-passengers wore a face mask compared with 47% (15/32) of control-passengers (OR 0, 95% CI 0–0.71). For the New York to Hong Kong flight, no case-passengers wore a face mask compared with 47% (15/32) of control-passengers (OR 0, 95% CI 0–0.71). Among control-passengers who used face masks, 4 did not use them during the New York to Vancouver trip, and 3 did not use them during the Vancouver to Hong Kong trip. Exposure to any lavatories or specific lavatories, talking with other passengers, moving around the aircraft, and reported hand hygiene during the New York to Hong Kong flight were not associated with being a case-passenger (Table). Reported handwashing was highly homogeneous among case- and control-passengers and was performed exclusively at each visit to the lavatory and by using the wet towel provided before meals. No one in the case and control groups had contacted with patients with ILI ≤1 week before the flight.

**Table:** Case–control analysis of potential risk or protective factors for 9 case-passengers infected with influenza A(H1N1)pdm09 virus and 32 control-passengers on a flight from New York, New York, to Hong Kong, China, May 2009*

Risk or protective factor	No response, no. (%)†			Response, no. (%)		OR (95% CI)‡	2-tailed p value‡ Case
Case	Control	Case	Control
Age, y						Age, y	
<20	0	0		4 (44)	4 (12)	<20	0
20–40	0	0		4 (44)	15 (47)	20–40	0
>40	0	0		1 (11)	13 (41)	>40	0
Male sex	0	0		5 (56)	15 (47)	Male sex	0
Chinese ethnicity	0	0		8 (89)	32 (100)	Chinese ethnicity	0
Flight from New York to Vancouver						Flight from New York to Vancouver	
Wearing mask	0	0		1 (11)	16 (57)	Wearing mask	0
Using lavatory 3	0	3 (11)		2 (22)	7 (28)	Using lavatory 3	0
Using lavatory 5	1 (11)	3 (11)		2 (25)	4 (16)	Using lavatory 5	1 (11)
Using lavatory 3 or 5	1 (11)	1 (3.6)		3 (38)	11 (41)	Using lavatory 3 or 5	1 (11)
Using lavatory 3, 4, 5, or 6	0	1 (3.6)		6 (67)	14 (52)	Using lavatory 3, 4, 5, or 6	0
Flight from Vancouver to Hong Kong						Flight from Vancouver to Hong Kong	
Wearing mask	0	0		0	15 (47)	Wearing mask	0
Using lavatory 3	0	2 (6)		5 (56)	7 (23)	Using lavatory 3	0
Using lavatory 5	1 (13)	2 (6)		1 (13)	5 (17)	Using lavatory 5	1 (13)
Using lavatory 3 or 5	1 (13)	0		5 (63)	12 (38)	Using lavatory 3 or 5	1 (13)
Using lavatory 3, 4, 5, or 6	0	0		7 (78)	20 (63)	Using lavatory 3, 4, 5, or 6	0
Flight from New York to Hong Kong						Flight from New York to Hong Kong	
Wearing mask	0	0		0	15 (47)	Wearing mask	0
Talking with other passengers	0	0		2 (22)	6 (19)	Talking with other passengers	0
Moving around airplane	0	0		1 (11)	3 (9.4)	Moving around airplane	0
Contact with patients with ILI	0	0		0	0	Contact with patients with ILI	0
Hand sanitation¶						Hand sanitation¶	
Washing hands when using lavatory	0	0		9 (100)	32 (100)	Washing hands when using lavatory	0
Cleaning hands before eating	0	0		8 (89)	29 (91)	Cleaning hands before eating	0
Among 9 case- and 17 control-passengers who did not wear masks during flight from Vancouver to Hong Kong							
Using lavatory 3	0	1 (6)		5 (56)	6 (38)	Using lavatory 3	0
Using lavatory 3 or 5	1 (13)	0		5 (63)	9 (53)	Using lavatory 3 or 5	1 (13)
Using lavatory 3, 4, 5, or 6	0	0		7 (78)	13 (76)	Using lavatory 3, 4, 5, or 6	0

*Of the 32 control-passengers on the flight from New York to Hong Kong, 28 boarded in New York and 4 boarded in Vancouver, British Columbia, Canada. OR, odds ratio; NA, not applicable; ILI, influenza-like illness; NE, not estimated. †No. case-passengers or control-passengers who did not answer the question. ‡By Fisher Exact test and StatXact 8 (*15*). §Difference among 3 age groups. ¶Always washed hands when using lavatory and always used wet towel before eating.

## Discussion

During this outbreak, influenza A(H1N1)pdm09 virus appeared to have been transmitted on a New York to Hong Kong flight. No other common time–place exposure could account for the point-source pattern. The most probable exposure period was during the New York to Hong Kong flight, in the Hong Kong airport, or during the Hong Kong to Fuzhou flight. Lack of cases in passengers or crew members on the Hong Kong to Fuzhou flight who were not on the New York to Hong Kong flight and the case in the Hong Kong resident suggested that exposure was not on the Hong Kong to Fuzhou flight or after landing in Fuzhou. Our results do not support exposure in New York before arrival at the airport, except that the estimated exposure period included the final 12 hours in New York. Exposure at common points in the airport in New York (e.g., at the check-in counter or security checkpoints) would have been brief and thus unlikely to lead to a high attack rate.

Furthermore, passengers did not wear masks at these points, and we would not have shown their protective effect. Before arrival at the airport, case-passengers were not together at the same place at the same time to account for the point-source pattern. For the 4 nonstop flights/day from New York airports to China during May 29–June 2, there were 4 confirmed influenza A(H1N1)pdm09 infections, which is equivalent to 0.2 infections/flight. Exposure in New York led to a prevalence of infection among passengers similar to the prevalence of influenza A(H1N1)pdm09 during the same week among the general population of New York. However, published surveillance estimates in the United States indicated that the 348 confirmed influenza A(H1N1)pdm09 virus infections reported in New York that week would be equivalent to a prevalence of 0.31%, which is similar to the previous estimate of <1 case among the passengers on the New York to Hong Kong flight (*16*).

This outbreak highlights the role of air travel in spread of influenza infections (*17**–**20*). All 9 infected passengers during the incubation period passed through airport fever and symptom screening, indicating that transmission on flights can escape detection. Also, 106 passengers on the New York to Hong Kong flight flew to other destinations and passed through different quarantine posts. In addition, an unknown number of the 91 passengers who traveled to Hong Kong continued into China by bus, ferry, train, and car through different quarantine posts. By the time we recognized the link to the New York to Hong Kong flight, passengers had dispersed and could not be traced. We estimate that 106 economy-class passengers, for whom risk for infection was 11%, traveled onward, potentially leading to dissemination of 12 infections to multiple sites.

The case-passengers were seated in 2 separate cabins of economy class. Previous investigations showed that increased risk for influenza in aircraft clustered within 2 rows in front of and behind a passenger with ILI (*18**–**21*). The source case-patient(s) might have been among the 106 transit passengers who were not screened in Hong Kong and who flew to other destinations and could not be traced. Without the source case-patient(s) being identified, we cannot explain the dispersed distribution, but we can offer some possibilities. There might have been ≥2 unrelated source case-passengers on the flight seated in each of the economy class cabins. A crew member serving economy class might have been infectious. However, all 14 crew members showed negative results when screened in Hong Kong. A common and frequently visited area such as a lavatory or food-service area might have been heavily contaminated with nasopharyngeal droplets from an infectious passenger. However, we did not find an association with lavatory use or general frequency of moving around the aircraft.

Airborne transmission in the airplane might be possible. Experiments and simulations show that particles <2 μm in diameter could be distributed widely, albeit at a low concentration, from a single source throughout an aircraft cabin (*22*). Influenza outbreaks in a train and an aircraft cabin with nonoperating air conditioning showed wide distribution of secondary cases, suggestive of airborne transmission (*23**,**24*). Infection from a fellow passenger should also have resulted in clustering from the much longer and closer exposure to respiratory droplet and aerosols during the 20-hour exposure during the flight.

Observational studies in hospitals, households, and community settings have shown a range of protective effects of face mask use against confirmed influenza, ILI, or respiratory infection (range 0%–74% reduction) (*25**–**34*). Several factors might explain the stronger effect observed in this outbreak. Exposure was for <24 hours in a confined space with limited activity of exposed persons. The other studies all involved days to months of exposure in the community or hospitals with free movement outside the immediate setting where face masks were used. Compliance with face mask use was probably greater among travelers on a single flight who were concerned about unpredictable health effects of the new virus. In 2 household studies, contacts were already exposed before the face mask was first worn (*26**,**29*). Only 2 of 7 other studies detected protection against confirmed influenza infection (*29**,**30*).

Extensive surveillance data for the United States showed that even at the peak of seasonal influenza transmission, <35% of persons with ILI had confirmed influenza (*35*). Other viruses causing ILI and having higher ratios of droplet transmission will lessen the observed epidemiologic effect of measures that protect against aerosol transmission. Face masks also have an unintended effect of reducing frequency of touching the mouth and nose and self-infection from contaminated hands. Accordingly, their protective effect, although suggestive, is not conclusive for airborne transmission of inhaled or inspired aerosols. Because long-distance air travel is a major route of dissemination of influenza virus (*17**,**18**,**36*), our findings regarding the effect of face mask use on flights should be evaluated further and considered for decreasing spread of influenza virus.

Hand hygiene has been recommended for preventing influenza transmission (*37*). In this outbreak, reported handwashing after lavatory use was universal and hand cleaning before meals was nearly universal for all passengers. Thus, we were unable to examine any effect of hand hygiene. However, hand hygiene would not have altered the effect of face mask use.

Direct experimentation and computer simulations indicate that N95 face masks should reduce the risk for airborne transmission of influenza virus by aerosols containing droplet nuclei (diameter <2 μm) in aircraft cabins by 90% (*38**–**40*). Less efficient face masks (e.g., surgical or medical) also decrease exposure to aerosols of droplet nuclei to a lesser (8–12 fold) degree than N95 masks (*36*), and they provide protection against larger droplets. We did not determine the type of mask worn by the passengers; presumably, individually acquired masks represented a mixture of N95 and other less efficient masks. Our findings are based on a small number of influenza infections, and an actual effectiveness of 90% is well within the confidence level of our estimate. The source case-person(s) of influenza virus on the flight might have taken a cough suppressant and might not have been actively coughing. If influenza virus had been expelled by normal breathing only, protection by an N95 mask for a 4-hour flight could approach 100% (*40*). Finally, infection from larger inspired or inoculated droplets from an infected person who actively circulated throughout the economy cabins could also explain the observed protection afforded by less-efficient mask types.

This investigation had several limitations. We lacked seating and illness information for 68% of the economy-class passengers on the New York to Hong Kong flight, among whom was probably the source case-passenger. The missing source case-passenger is also a gap in the evidence that transmission occurred on the flight. We were unable to determine the outcome of passengers and crew who disembarked in Vancouver and whether transmission occurred during 1 or both legs of the flight. Types of face masks used were unknown. With only 9 cases in 25% of the passengers, our case–control study had poor sensitivity.

In summary, this outbreak probably resulted from a common source exposure to influenza A(H1N1)pdm09 virus on the New York to Hong Kong flight. Wearing a face mask was associated with a decreased risk for influenza acquisition during this long-duration flight. Border entry screening did not detect case-passengers during the influenza incubation period. We recommend a more comprehensive intervention study to accurately estimate the protective effect of face masks for preventing influenza virus transmission on long-distance flights.
